# Assessment of Changes in Udder Half Defects over Time in Non-Dairy Ewes

**DOI:** 10.3390/ani13050784

**Published:** 2023-02-21

**Authors:** Mandefrot M. Zeleke, Kate J. Flay, Paul R. Kenyon, Danielle Aberdein, Sarah J. Pain, Anne L. Ridler

**Affiliations:** 1 School of Veterinary Science, Massey University, Private Bag 11222, Palmerston North 4410, New Zealand; 2 School of Veterinary Medicine, Wolaita Sodo University, Wolaita Sodo P.O. Box 138, Ethiopia; 3 Department of Veterinary Clinical Sciences, City University of Hong Kong, Kowloon, Hong Kong SAR, China; 4 School of Agriculture and Environment, Massey University, Private Bag 11222, Palmerston North 4410, New Zealand

**Keywords:** change over time, culling, diffusely hard udder, non-dairy ewe, palpable udder defect, relative risk, and udder lump

## Abstract

**Simple Summary:**

Two studies were undertaken to assess the changes in palpable udder half defect (hard, lump, or normal) over time and the prediction of future occurrence in a non-dairy breed (Romney) of ewes. In the first study, a standardized udder scoring method was applied at pre-mating, pre-lambing, docking, and weaning. The second study assessed the udder half defect changes in the first six weeks of lactation. The results show that a higher occurrence of diffusely hard udder halves were observed at either pre-mating or docking, while a higher occurrence of udder half lumps were observed at docking and weaning. Udder halves detected with a diffuse hardness or lumps of various sizes at pre-mating were more likely to have a palpable udder half defect (either hard or lump) at pre-mating, docking, or weaning, within the same year, or at pre-mating in the following year. Udder half defect status was highly variable in early lactation; however, overall, the number of defective udder halves decreased with lactation. Thus, these findings show that the risk of future occurrence of a defect was higher in udder halves previously identified with either hard or lump and, therefore, ewes with diffusely hard udder halves or udder half lumps should be culled and not retained for breeding.

**Abstract:**

A total of 1039 non-dairy breed (Romney) ewes were enrolled in two studies to assess the changes in udder half defect status (hard, lump, or normal) over time and to predict the risk of future udder half defect occurrence. In the first study (study A), udder halves of 991 ewes were assessed utilizing a standardized udder palpation method and scored four times a year, for two successive years (pre-mating, pre-lambing, docking, and weaning). The second study (study B) assessed the udder halves at pre-mating, and at six weekly intervals in the first six weeks of lactation in 46 ewes that had defective and normal udder halves. Udder half defect change over time was visualized via lasagna plots, and multinomial logistic regression was used to predict the risk or probability of udder half defect occurrence. In the first study, the highest occurrence of udder halves categorised as hard was observed at either pre-mating or docking. Udder halves categorised as lump had their highest occurrence at either docking or weaning. Udder halves detected with a defect (hard or lump) at pre-mating were more likely (RRR: 6.8 to 1444) to be defective (hard or lump) at future examinations (pre-lambing, docking, or weaning) within the same year or pre-mating the following year, compared to udder halves categorised as normal. In the second study, the change of udder half defect type over the first six weeks of lactation was variable. However, it was observed that the udder half defects, particularly udder halves categorised as hard, decreased during lactation. Failure to express milk in udder halves in early lactation was associated with a higher occurrence and persistency of udder half defects. In conclusion, the occurrence of diffuse hardness or lumps in an udder half changed over time, and the risk of future occurrence of a defect was higher in udder halves previously categorised as either hard or lump. Hence, it is recommended that farmers identify and cull ewes with udder halves categorised as hard and lump.

## 1. Introduction

Poor udder health can have serious impacts on the welfare of suckling lambs and reduce the longevity of ewes within the flock [1,2]. In non-dairy ewes, defective udders are associated with lower lamb survival and decreased lamb live weight gain during the pre-weaning period [3,4]. Udder defects are abnormalities of the udder, the causes of which can range from non-mechanical injury to infectious agents [5,6]. Several terminologies have been used to describe defective udders such as “impaired udders”, “imperfect udder”, “unsound udder”, “faulty udder”, “udder with abnormality” and “defective udder” [4,5,7,8]. Udder defects include abnormal secretion, inflammation, nodules or mammary masses, diffuse hardness, cysts, fibrosis, teat occlusion, teat canal cord formation, lumps, and ruptured abscesses on the udder [4,8,9,10,11,12,13,14].

Clinically, ewe udder defects can be detected by physical palpation and, on occasion, visually [8,15]. Standardized udder assessment is a useful tool to identify defective udders of ewes to provide timely selective treatment or make culling decisions [12,16]. Udder defect assessment in non-dairy ewes usually takes place either at weaning or prior to mating when farmers are selecting ewes for the next breeding season [8]. More than 75% of New Zealand farmers reportedly assess their ewes’ udders at least once a year, which is assumed for the purpose of making culling decisions [17]. Ridler, et al. [18] recommended that ewe udders are examined at least four to six weeks post-weaning as issues not identified at weaning can be more easily identified.

Palpable udder defects such as hardness within the udder and various-sized lumps have been reported during pregnancy, lactation, and/or the dry period in ewes [12]. The hard udder has been described as a diffuse hard consistency of the udder upon palpation [4,8]. Udder lumps (abscesses) have been described by several authors and have been reported to show phenotypic diversity in size, consistency, and location upon clinical and post-mortem examination of udders [4,8,11,18,19].

Repeated udder examinations of the same flock of ewes within a season in New Zealand [4] and across two seasons in the United Kingdom [12] both showed phenotypic diversity and a varying percentage of udder defects on consecutive examinations. Ridler, et al. [18] also reported changes in udder defects across two visits, where the first visit was on the day of weaning followed by the second visit four to six weeks later. In that study, ewes with hard udders at the first visit changed to normal or lump or remained hard while there was a small number of normal udder halves at the first visit that were subsequently found to have defects at the second visit. Bruce, et al. [20] reported that “hard udder” typically occurred shortly after lambing and then appeared to regress, with involution of the gland occurring within 3–4 weeks of initial lesion detection. In addition, Peterson, et al. [21] reported hard udders identified by palpation three weeks post-weaning had changed to normal palpation scores several months past weaning. Smith, et al. [11] hypothesized that the phenotypic diversity of palpable udder lumps (abscesses) could be due to the maturation cycle of the abscesses. Combined these studies imply that udder defects can change over time. Further, Grant, et al. [12] reported that ewes that were identified with palpable udder defects were three to five times more likely to have udder defects identified in the future.

Understanding the dynamic nature of palpable udder defects over time could provide important information regarding when higher rates occur and the best possible time for an intervention. Additionally, such studies could provide reliable information regarding the appropriate culling of affected ewes, by predicting the likelihood of recurrence within the same or the following year/season. Therefore, the objectives of these studies were: to assess the changes in udder half defect status over time, both over two full years and during a single lactation; and to predict the risk of future udder half defect occurrence.

## 2. Materials and Methods

This manuscript utilized data from two studies. Study A was undertaken under commercial conditions and utilised longitudinal data gathered over a two-year period from 2017 to 2018. Study B used data from 48 ewes with a history of either udder defects or normal udders. In study B, all ewes were presented for machine milking once followed by hand stripping according to Peterson, et al. [22] in the first six weeks of lactation in 2019. In both studies, lambs were with their dams until weaning.

### 2.1. Study A: Ewe Selection and Management

The study comprised the entire cohort of 2013 (n = 590) and 2014-born (n = 391) replacement ewes at Massey University Riverside farm (n = 981 on 17 February 2017, at pre-mating, Wairarapa, New Zealand). All ewes had lambed at least once prior to joining the study. Ewes were ear tagged with visual (VID; Allflex, Palmerston North, New Zealand) and electronic (EID; Allflex, Palmerston North, New Zealand) identification tags. In autumn each year (March), rams were introduced to the ewes for breeding at a ratio of approximately 1:150 for two oestrus cycles (34 days). Transabdominal ultrasonography was undertaken by a commercial practitioner during mid-pregnancy to diagnose pregnancy status and determine the number of foetuses. Throughout the study period, ewes were managed under commercial conditions with individual feeding decisions at the flock level, being at the discretion of the farm manager. The predominant pastures were ryegrass (*Lolium perenne*) and white clover (*Trifolium repens*). At pre-lambing (set-stocking), approximately 10 days before the expected start of lambing, the ewes were divided into three groups accounting for expected litter size (singleton-, twin- and triplet-bearing). Pasture measurements were not taken, but the ewes were allocated into different paddocks for lambing at a rate of approximately 7 to 12 ewes per hectare, based on the expected litter size. Lambing took place in spring and during lambing ewes were monitored twice daily to match lambs to their dams. Lambs were given an electronic identification tag and relevant information on newborn lambs was recorded: dam ID, date of birth, sex, birth rank, and birth weight. Ewes were culled based on routine farm practices such as feet and teeth; however, ewes were not culled based on udder health. Non-pregnant ewes were also culled each year. In addition, throughout the study, ewes that required euthanasia on welfare grounds were euthanised.

### 2.2. Study B: Ewe Selection and Management

In December 2018, 80 ewes aged 5–6 years were obtained from Massey University’s Riverside farm, Wairarapa, New Zealand. These ewes were selected based on udder traits evaluated during Study A. Sixty of these ewes had one or more udder half defects (hard and/or lump) or a history of udder half defects over the previous two years whereas 20 ewes had never been recorded as having an udder half defect. After selection, these ewes were moved to Massey University’s Keeble farm, which is located 5 km southeast of Palmerston North, New Zealand. Ewe selection, reproductive management, pregnancy diagnosis, culling and lambing, and management were described in our previous publication [23].

### 2.3. Body Condition Scoring (BCS) and Live Weight

In both studies, BCS was assessed by an experienced assessor by palpating the lumbar region and feeling the vertical (spine) and horizontal processes along the loin area. The body condition scoring system was a 5-point system (1–5, where: 1 = thin and 5 = obese) assessed at 0.5 intervals [24,25]. Ewes were weighed by using static digital weighing scales (model XR5000, Tru-Test Group, Auckland, New Zealand) to the nearest 0.2 kg at each body condition scoring event. Body condition scoring and live weight measurement of each ewe were also assessed on udder scoring days.

### 2.4. Udder Scoring

Ewe udders were assessed by trained and experienced assessors with ewes restrained by experienced sheep handlers in a sitting position. Udder halves were scored from 1–7 using the system described by Griffiths, et al. [4]; however, the scores were then collapsed down to three categories: normal (scores 1 and 2); hard (score 7); and lump (scores 3–6).

In Study A, udders were assessed four times per year for two consecutive years (2017–2018) at pre-mating (February), pre-lambing (September), docking (3–8 weeks after lambing, November), and weaning (December). These time points were selected as they represent key management events when farmers traditionally interact with individual ewes. Four trained operators undertook the udder palpations in Study A.

Previous studies on udder defects in ewes reported a higher occurrence in early lactation compared to weaning and post-weaning periods [20,21]. According to the same authors, a change in udder defect status was observed shortly after lambing. Therefore, in Study B, udders were assessed at pre-mating and then once weekly in the first six weeks of lactation beginning from 4–10 days after parturition. In Study B a single operator undertook all udder palpations to avoid potential inter-operator variation.

### 2.5. Data Analysis

In Study A, all 981 ewes (1962 udder halves) in 2017, 769 ewes (1538 udder halves) in 2018, and 704 ewes (1408 udder halves) in both years were included in the analysis. In Study B, 48 ewes (92 udder halves) were assessed weekly in the first six weeks of lactation. However, in both studies, only udder halves with complete data for all scoring events within a plot were considered for the lasagna plots.

#### 2.5.1. Descriptive Analysis—Lasagna Plots

To analyse the dynamic nature of udder defects the lasagna plot method [26,27] was identified as the most suitable option. Lasagna plots allow visualization of the changes in the defect category (hard, lump, or normal) of udder halves over time (for the different udder scoring events). Within the plots, each bar shows a different udder scoring event (i.e., time) while the different colours within each bar represent the defect categories. The data in the table at the top of the plot designates the percentage of each udder half defect category at each event, which corresponds to the percentage of each colour at each event. Change in defect category over time of each udder half can be tracked by following longitudinal transitions across the udder scoring events of stacked bars. A change in the colour of each udder-half category at a different time event represents a change in the udder-half category. The lasagna plots were created using SAS Statistical software Version 9.4 (SAS Institute Inc., Cary, NC, USA) utilizing the methodology of Jones, et al. [27].

Three lasagna plots were created using udder half data from Study A: (i) four udder examination events in 2017 comprising 1860 udder halves (930 ewes), (ii) four udder examination events in 2018 comprising 1428 udder halves (714 ewes), and (iii) all eight udder examination events across 2017 and 2018 comprising 1408 udder halves (704 ewes).

Four lasagna plots were created for Study B, (i) pre-mating and six udder examination events during their single lactation which comprised data from udder halves from which milk was expressed during the once-weekly milking events (43 udder halves), (ii) pre-mating and six udder examination events during lactation which comprised data from udder halves from which milk was not expressed (37 udder halves), (iii) pre-mating and six udder examination events during their single lactation which comprised data from udder halves from which had a previous history of udder half defect (58 udder halves), (iv) pre-mating and six udder examination events during lactation which comprised data from udder halves from which milk had no previous history (i.e., normal) of udder half defect (22 udder halves). Udder halves that did not express milk were defined as those udder halves from which no milk was expressed from at least four of the six weekly milking events and expressed less than 100g/day in the rest, otherwise, an udder half was considered as expressed milk.

#### 2.5.2. Multinomial Logistic Regression

Study A. A series of multinomial logistic regression models were developed utilising data from 1962 udder halves in 2017, and 1562 udder halves in 2018 to predict the relative risk ratio of udder half defect status (hard, lump, or normal) at pre-lambing, docking, and weaning for the 2017 year and the 2018 year, based on the udder half defect status at pre-mating of each respective year being analysed. Two models were fitted from data of the 1426 udder halves that were measured across both years to predict the relative risk ratio of the udder half defect status at pre-mating in 2018 based on pre-mating and weaning data in 2017. Birth rank (singleton [1] or twin [2]), rearing rank (no lambs reared [0], reared a single lamb [1] or reared twin lambs [2]), udder half (right/left), age of ewes (2013 or 2014 year of birth) and ewe BCS and bodyweight at premating were included as covariates. For all the models developed, the probability of each udder half defect category at each udder scoring event was extracted.

Study B. Several multinomial logistic regression models were fitted to assess weekly transitional probability using data from 48 ewes during the first six weeks of lactation, conditionally on the preceding udder palpation event (e.g., Day 7 udder defect status was used to predict Day 14 udder defect status, Day 14 for Day 21, and so forth for the six examinations during lactation). Birth rank (singleton [1] or twin [2]), rearing rank (no lambs reared [0], reared a single lamb [1], or reared twin lambs [2]), udder half (right/left) and udder half milk expression status (yes/no) were included as covariates. A separate analysis, using the same model structure, was undertaken to predict the probability of each udder half defect category in the six examination events during lactation (Day 7 to Day 42) based on the pre-mating udder half defect status of the ewes at mating in 2019.

All analyses were performed using a multinomial logistic regression approach with a ‘*multinomial*’ function from the ‘*net*’ package in R statistical software version 3.6.3 [28]. Interactions between variables were tested. The model development process was based on the backward selection of the independent variables mentioned. The goodness of fit of the models was tested for significance by the likelihood ratio test (also called model chi-square). Variance inflation factor was applied to assess the multicollinearity while the goodness of fit was assessed by McFadden’s pseudoR^2^.

## 3. Results

### 3.1. Study A: Lasagna Plots of Udder Half Defects in 2017 and 2018

**Figure 1:**
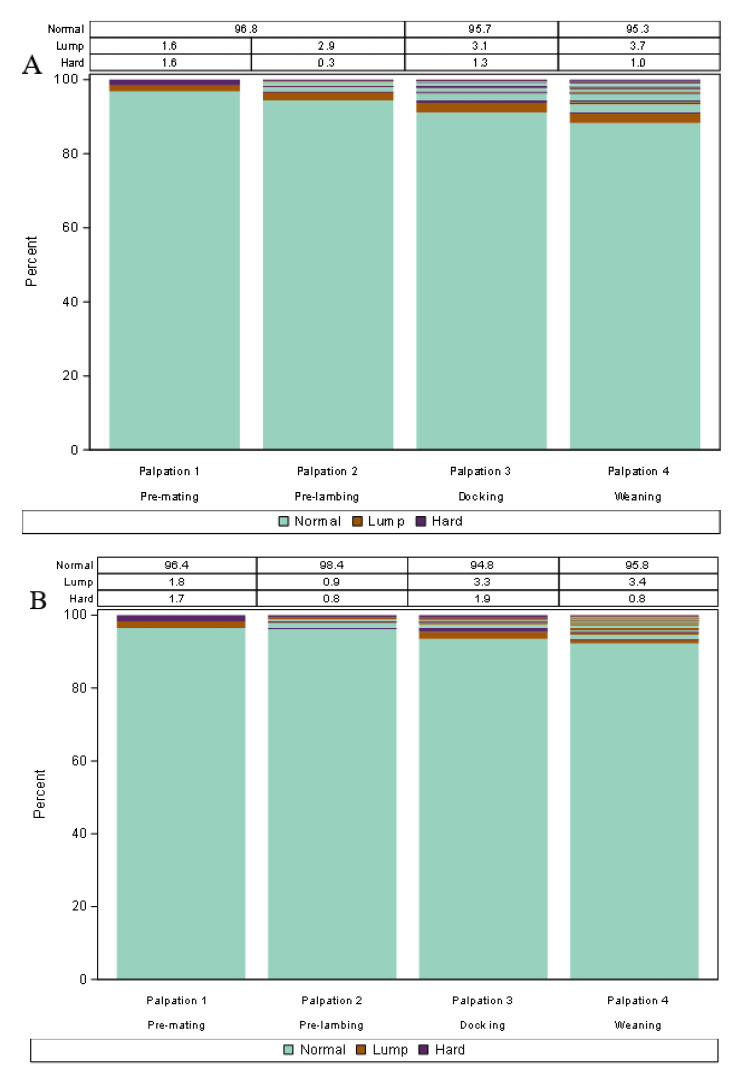

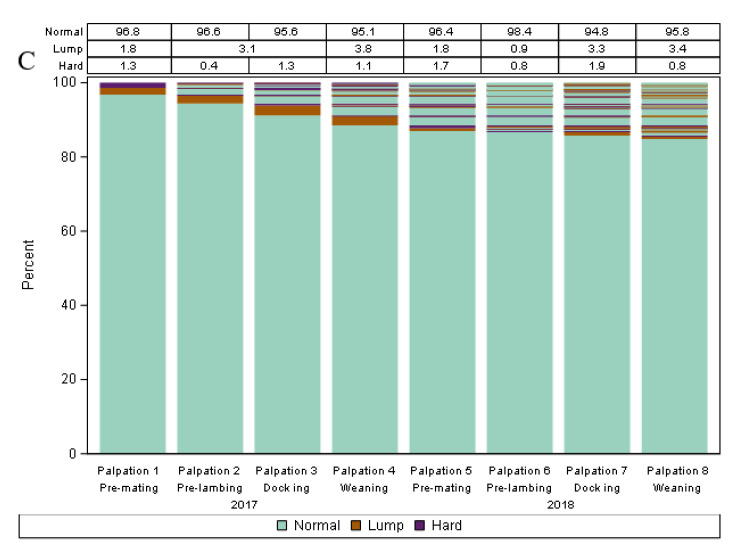
Lasagna plot of palpable udder half defects (hard, lump, and normal) from non-dairy Romney ewes identified during udder examination events occurring at key management times (pre-mating, pre-lambing, docking, and weaning; Study A). (**A**) Lasagna plot of 1860 udder halves (930 ewes) in the year 2017; (**B**) 1428 udder halves (714 ewes) in the year 2018; (**C**) 1408 udder halves (704 ewes) from 2017 and 2018 combined. Note: Within each plot, each bar shows a different udder half scoring event (i.e., time) while the different colours within each bar represent the defect categories. The data in the table at the top of each plot designates the percentage (%) of each udder half defect category at each event, which corresponds to the percentage of each colour at each event. Change in defect category over time of each udder half can be tracked by following longitudinal transitions across the udder scoring events of stacked bars.

Overall, at least 94.8% of udder halves were normal at each individual udder examination event, although new udder half defects were detected at each additional examination event from those udder halves previously categorised as normal (Figure 1). Amongst udder halves that had defects (hard or lump) or those that developed defects, the lasagna plots demonstrate that defects either remained the same over time, changed to normal, or changed to another defective category, i.e., all possible changes occurred although only a relatively small proportion of ewes had or developed defects. Only six (0.3%) udder halves in 2017 and 16 (1.0%) udder halves in 2018 stayed defective (remained in the same category or changed to another defective category) at all udder examination events within each year. However, none of the udder halves remained defective in all eight udder examinations throughout both years.

**Figure A1:**
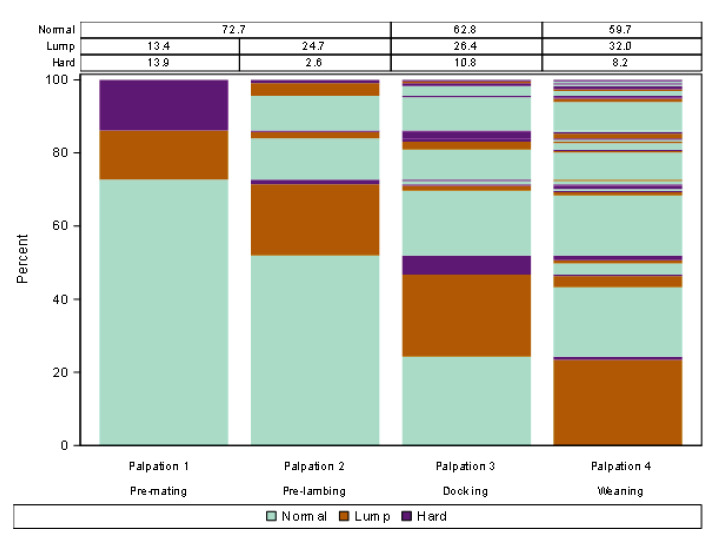
Lasagna plot of palpable udder half defects (hard, lump and normal) from non-dairy Romney ewes identified during udder examination events occurring at key management times (pre-mating, pre-lambing, docking, and weaning; Study A). Lasagna plot of 231 udder halves in year 2017, excluding udder halves observed with no udder defect on all occasions. Note: Within each plot, each bar shows a different udder half scoring event (i.e., time) while the different colours within each bar represent the defect categories. The data in the table at the top of each plot designates the percentage (%) of each udder half defect category at each event, which corresponds to the percentage of each colour at each event. Change in defect category over time of each udder half can be tracked by following longitudinal transitions across the udder scoring events of stacked bars.

**Figure A2:**
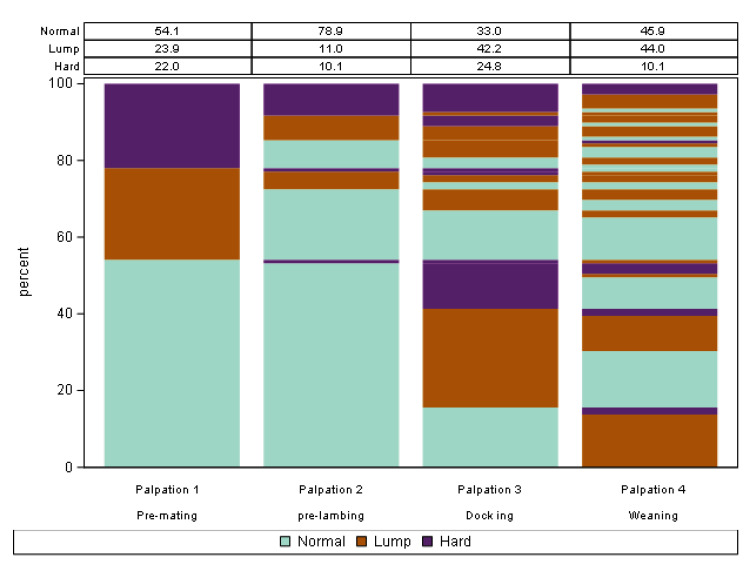
Lasagna plot of palpable udder half defects (hard, lump, and normal) from non-dairy Romney ewes identified during udder examination events occurring at key management times (pre-mating, pre-lambing, docking, and weaning; Study A). Lasagna plot of 109 udder halves in the year 2018, excluding udder halves observed with no udder defect on all occasions. Note: Within each plot, each bar shows a different udder half scoring event (i.e., time) while the different colours within each bar represent the defect categories. The data in the table at the top of each plot designates the percentage (%) of each udder half defect category at each event, which corresponds to the percentage of each colour at each event. Change in defect category over time of each udder half can be tracked by following longitudinal transitions across the udder scoring events of stacked bars.

**Figure A3:**
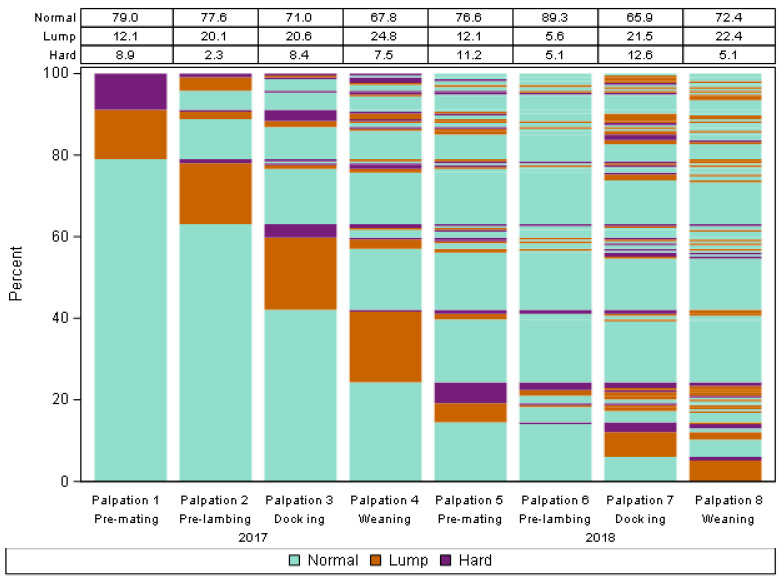
Lasagna plot of palpable udder half defects (hard, lump, and normal) from non-dairy Romney ewes identified during udder examination events occurring at key management times (pre-mating, pre-lambing, docking, and weaning; Study A). Lasagna plot of 214 udder halves in the years 2017 and 2018, excluding udder halves observed with no udder defect on all occasions. Note: Within each plot, each bar shows a different udder half scoring event (i.e., time) while the different colours within each bar represent the defect categories. The data in the table at the top of each plot designates the percentage (%) of each udder half defect category at each event, which corresponds to the percentage of each colour at each event. Change in defect category over time of each udder half can be tracked by following longitudinal transitions across the udder scoring events of stacked bars.

In both years, the percentage of udder halves categorised as hard was higher at the pre-mating examination compared with pre-lambing (late-pregnancy). The percentage increased again at docking before slightly decreasing at weaning (Figure 1A,B). Udder halves, categorised as lumps, were more commonly identified during lactation (i.e., identified at docking and weaning) compared to during the non-lactation periods (i.e., identified at pre-mating and pre-lambing; Figure 1A,B). A comparison of the same examination periods across years showed that the percentage of udder halves that were defective was higher at pre-mating (0.4%) and docking (0.9%) in 2018 compared to 2017 (Figure 1C). From eight udder examination events over the two-year period, the highest percentage of udder half defects was observed at docking 2018. Additional plots are included in Figure A1, Figure A2 and Figure A3, corresponding to Figure 1A–C, respectively, which show changes in the udder half defect category over time for those udder halves with a defect on at least one occasion.

### 3.2. Study A: Relative Risk Ratios of Udder Half Defects at Pre-Lambing, Docking, and Weaning in 2017 and 2018 Based on Pre-Mating Status

If an udder half was categorised as hard at pre-mating, it was more likely to be categorised as hard at pre-lambing, docking, and weaning in both 2017 (Table 1) and 2018 (Table 2) compared to an udder half that was categorised as normal at pre-mating. The relative risk ratios (RRR) ranged from 14 to 1465 (*p* < 0.05) when other variables in the model were held constant. If an udder half was hard at pre-mating (within each respective year), the relative risk of it being categorised as a lump at the following pre-lambing in 2017 and pre-lambing, docking, and weaning in 2018 was higher (RRR range 14 to 1465 times) compared to an udder half that was categorised as normal at pre-mating while keeping all other variables constant.

Udder halves categorised as a lump at pre-mating (within each year) were more likely (*p* < 0.05) to be categorised as hard at pre-lambing, docking, and at weaning in 2017 and at pre-lambing and docking in 2018, compared with udder halves that had been categorised as normal at pre-mating, when other variables were held constant in the model (RRR range 6 to 67, Table 1 and Table 2). In addition, udder halves categorised as lumps at pre-mating were more likely to be categorised as lumps at pre-lambing, docking, and weaning in both 2017 and 2018, compared to udder halves that had been categorised as normal at pre-mating, when other variables held constant in the model (RRR range from 6 to 333, Table 1 and Table 2).

Among the covariates considered for models examining udder half defects across key management times, rearing rank was the only covariate that significantly (*p* < 0.05) influenced udder half defects and only at docking in both years. A rearing rank of zero (no lambs suckling) was associated with a higher relative risk of an udder half being categorised as a lump at docking in 2017 and both hard or lump at docking in 2018, compared with the reference rank of one lamb suckling. In both 2017 and 2018, ewes that reared twins were less likely to have udder halves categorised as hard (OR = 0.2, *p* < 0.05) compared to the reference of ewes that reared single lambs (Table 1 and Table 2).

### 3.3. Study A: Predicted Probabilities of Udder Half Defects at Pre-Lambing, Docking, and Weaning in 2017 and 2018 Based on Pre-Mating Status

For udder halves categorised as normal at pre-mating, the vast majority remained normal at pre-lambing, docking, and weaning across both years (predicted probability (Pp) of 0.96–0.99, Table 3). In both years, the probability was highest at pre-lambing followed by docking and then weaning. For udder halves that were categorised as normal at pre-mating, the Pp that they would subsequently change to hard or lump was very low in both years (i.e., less than 0.036, Table 3).

For those udder halves that were categorised as hard at pre-mating, the Pp of remaining hard or changing to hard was variable between years and time points; however, it was higher in 2018 than in 2017. In contrast, the Pp of these udder halves being categorised as normal ranged from 0.73 to 0.83 in 2017 but was much lower in 2018 (Table 3).

For those udder halves that were categorised as lumps at pre-mating, the Pp of changing to normal was lowest at docking in both 2017 and 2018 (Table 3). The Pp of udder halves categorised as lump changing to hard was higher in 2017 compared to 2018, but the highest probability was observed at docking in both years. The Pp of udder halves categorised as lump remaining as lump ranged from 0.069 to 0.296.

### 3.4. Study A: Relative Risk Ratio of Udder Half Defects at Pre-Mating 2018 Based on Weaning and Pre-Mating 2017

If an udder half was categorised as defective (hard or lump) at pre-mating in 2017, the RRR of it still being categorised as defective (hard or lump) at pre-mating in 2018 was higher (RRR range 6.6 to 15.4, *p* < 0.05) compared with udder halves categorised as normal at pre-mating in 2017 (Table 4). Keeping all other covariates constant, for udder halves categorised as hard at weaning in 2017, the RRR of being categorised as hard or as a lump at pre-mating in 2018 was 141 and 53 times higher (*p* < 0.05) respectively, compared with an udder half that was categorised as normal at weaning in 2017. If an udder half was categorised as a lump at weaning in 2017, the RRR of it being categorised as hard or lump at pre-mating 2018 was 6 and 16 times higher (*p* < 0.05) respectively, compared with an udder half that was categorised as normal at weaning in 2017 (Table 4).

### 3.5. Study B: Lasagna Plots of Udder Half Defects at Pre-Mating and Day 7 to 42 of Lactation in Udder Halves with or without a Previous History of Udder Defect

**Figure 2:**
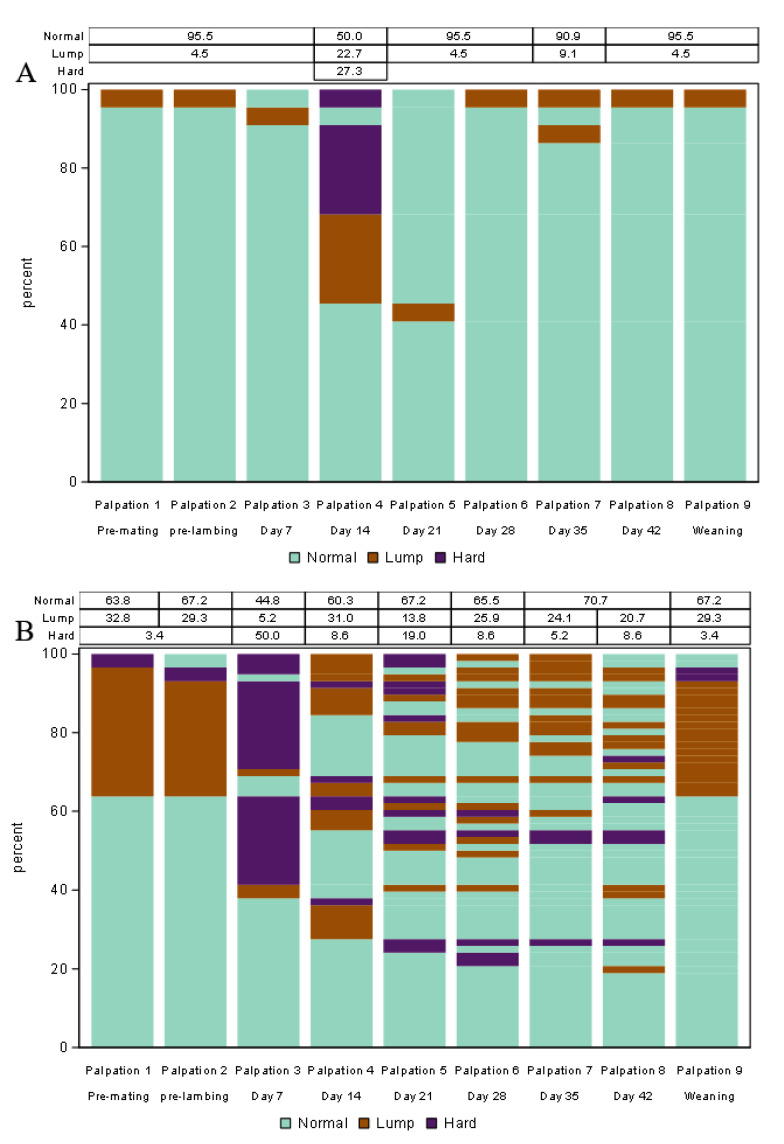
Lasagna plot of palpable udder half defects (hard, lump, and normal) in the first six weeks of lactation in non-dairy Romney ewes (Study B). Udder examination was undertaken at weekly intervals from 22 udder halves that had no history of defects in the previous two years (2017 and 2018); (**A**) and from 58 udder halves that had a history of defects (**B**). Note: Within each plot, each bar shows a different udder scoring event (i.e., time) while the different colours within each bar represent the defect categories. The data in the table at the top of the plot designates the percentage of each udder half defect category at each event, which corresponds to the percentage of each colour at each event. Change in defect category over time of each udder half can be tracked by following longitudinal transitions across the udder scoring events of stacked bars. Six ewes that missed the udder examination on Day 42 were excluded from the plot.

From a total of 22 udder halves that had no history of udder half defects in the previous two years (2017 to 2018), only 9 (41%) udder halves were found to be normal throughout all examinations. In contrast, in udder halves that had a history of udder half defects, 19% exhibited no udder half defects throughout all udder examinations. Udder half defects appeared to be highly variable with multiple changes over time, at pre-mating and during the first six weeks of lactation (Figure 2), particularly for those udder halves that had a previous history of defects (Figure 2B). The percentage of udder half defects, and their persistence, appeared to be higher in udder halves that had a previous history of defects (Figure 2B) compared with those that had not (Figure 2A).

From a total of 22 udder halves that had no defect history, only one udder half (4.5%) was categorised as a lump on Day 7 of lactation while the rest were categorised as normal (Figure 2A). The number of udder halves that had no defect history and were categorised as hard was highest on Day 14, and this was the only day that any of these 22 udder halves were categorised as hard. All udder halves categorised as hard on Day 14 had changed to normal by Day 21 and this persisted until Day 42; however, two (9%) udder halves changed to lump on Day 35. In udder halves with no defect history, only one new defect was observed on Day 35 and no new defect was observed in the following weeks.

From a total of 62 udder halves that had a previous history of defect, the percentage that was categorised as normal over six weeks of lactation appeared to be lower (45–71%, Figure 2B) compared with those that had no defect history (50–95%, Figure 2B). Fifty percent of udder halves that had a previous defect history were categorised as hard on Day 7, and over subsequent weeks, these udder halves changed category, such that no udder half remained consistently hard at all examinations. As the days in lactation increased, the percentage of udder halves categorised as hard decreased substantially. The percentage of udder halves that had a previous history of defects, and were subsequently categorised as lump, were higher on Day 14 compared with Day 7. At subsequent examinations, the percentage categorised as lump fluctuated over the weeks.

### 3.6. Study B: Lasagna Plots of Udder Half Defects at Pre-Mating and Day 7 to 42 of Lactation in Udder Halves That Did and Did Not Express Milk

**Figure A4:**
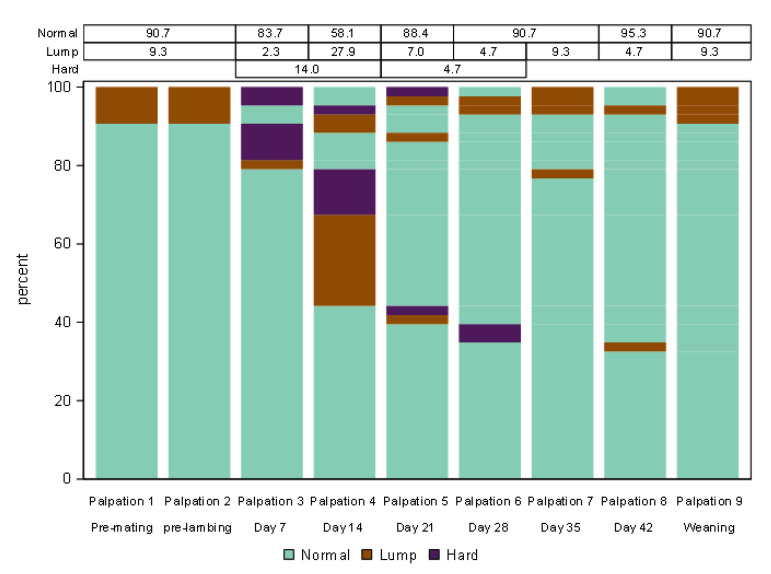
Lasagna plot of palpable udder half defects (hard, lump, and normal) in the first six weeks of lactation in non-dairy Romney ewes (Study B). Udder examination was undertaken at weekly intervals from 43 udder halves that did express milk. Note: Within each plot, each bar shows a different udder scoring event (i.e., time) while the different colours within each bar represent the defect categories. The data in the table at the top of the plot designates the percentage of each udder half defect category at each event, which corresponds to the percentage of each colour at each event. Change in defect category over time of each udder half can be tracked by following longitudinal transitions across the udder scoring events of stacked bars. Note that an udder half that did not express milk refers to an udder half that did not express milk at all on at least four occasions and expressed less than 100 g/day on the other two occasions.

**Figure A5:**
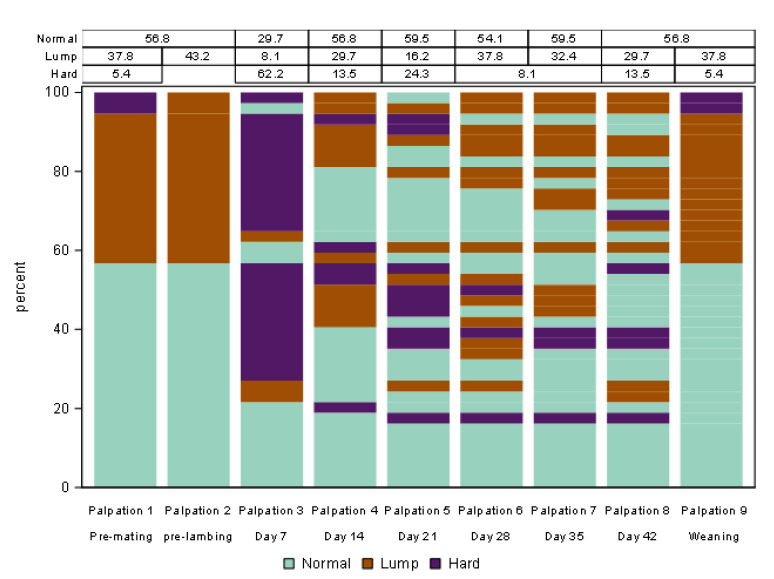
Lasagna plot of palpable udder half defects (hard, lump, and normal) in the first six weeks of lactation in non-dairy Romney ewes (Study B). Udder examination was undertaken at weekly intervals from 37 udder halves that did not express milk. Note: Within each plot, each bar shows a different udder scoring event (i.e., time) while the different colours within each bar represent the defect categories. The data in the table at the top of the plot designates the percentage of each udder half defect category at each event, which corresponds to the percentage of each colour at each event. Change in defect category over time of each udder half can be tracked by following longitudinal transitions across the udder scoring events of stacked bars. Note that an udder half that did not express milk refers to an udder half that did not express milk at all on at least four occasions and expressed less than 100 g/day on the other two occasions.

Milk expression status (yes/no) had a significant (*p* < 0.05) effect on the udder half defect. Compared to normal udder halves, those udder halves that did not express milk had higher RRR of being hard (OR = 7.9–35.4) on Days 14 and 21 and higher RRR of being lump (OR = 6–26, *p* < 0.05) on all six occasions during lactation compared to the normal udder halves. Except for one udder half, all udder halves that had no history of defect expressed milk whereas only 35% of udder halves that had a previous history of udder half defect expressed milk. Plots were made for udder halves that did express milk (Figure A4) and those that did not (Figure A5).

### 3.7. Study B: Weekly Transitional Probability of Udder Half Defects from Day 7 to 42 of Lactation

**Figure 3:**
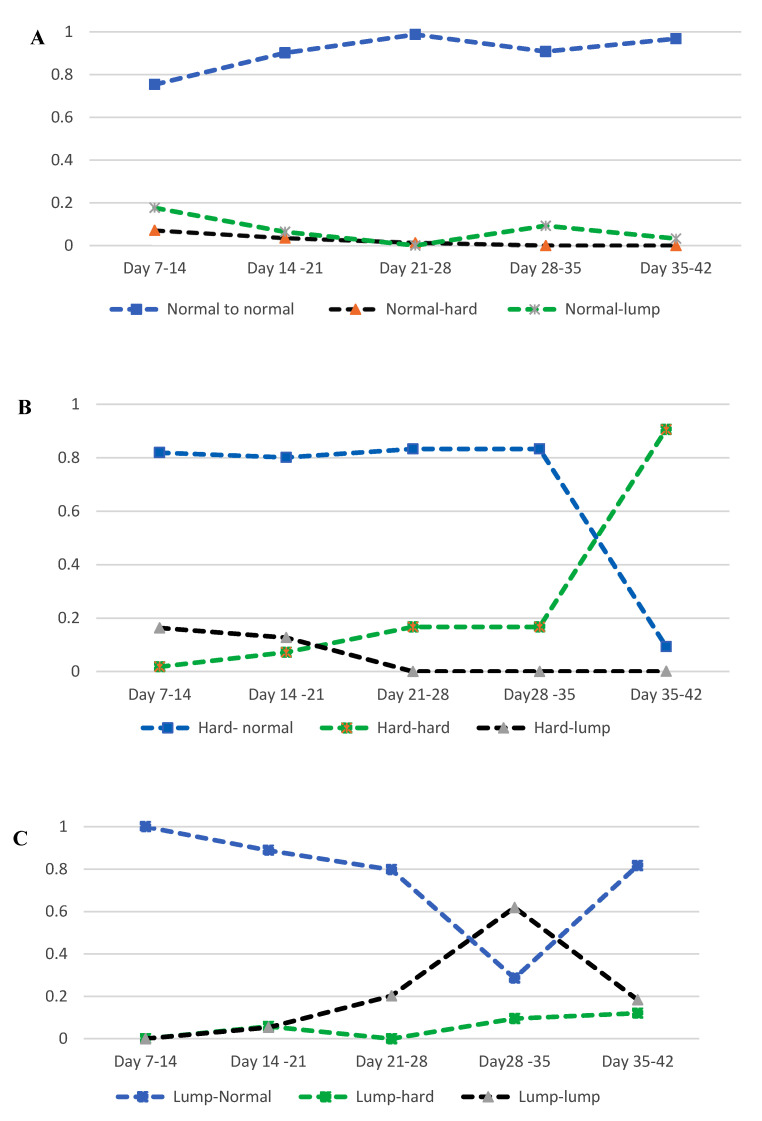
Line graph of the transitional probability of udder half defects in the first six weeks of lactation from 92 udder halves (from 46 non-dairy Romney ewes) in 2019 (Study B). (**A**) Transitional probability of udder half defect from preceding normal state; (**B**) Transitional probability of udder half defect from preceding hard state; (**C**) Transitional probability of udder half defect from preceding lump state. Probabilities were predicted conditionally on the preceding udder palpation event (e.g., Day 7 udder defect status was used to predict Day 14 udder defect status, Day 14 for Day 21, and so forth for the six examinations during lactation).

In the first six weeks post-lambing, weekly udder half defect category transitions (from Day 7 to 14, from Day 14 to 21, and so on) identified that all possible transitions across categories occurred (i.e., normal to normal; normal to hard or lump; hard or lump to normal; hard or lump to hard or lump, Figure 3). The probability of weekly transition of udder halves categorised as normal remaining normal increased as days in lactation advanced from Day 7 to 42. In contrast, the transitional probability of a normal udder half changing to an udder half categorised as either hard or lump declined (Figure 3A).

The weekly transitional probability of udder halves categorised as hard changing to normal was high (>0.8) during the first three weeks (Day 7 to 21) of lactation and remained steady until it declined from Day 28 (Figure 3B). The weekly transitional probability of an udder half categorised as hard that remained hard, increased as lactation advanced from Day 7 to 42, whereas the probability of transition from hard to lump slightly decreased. The weekly transitional probability of udder halves categorised as lump transitioning to normal declined from day 7 to Day 35 but then started to rise at Day 42 (Figure 3C). In contrast, the weekly transitional probability of udder halves categorised as lump remaining as lump increased from Day 7 to day 35 and then declined at Day 42. The probability of an udder half categorised as a lump transitioning to hard was variable among the udder scoring days, but it was generally low (Pp < 0.09).

### 3.8. Study B: Predicted Probability (Pp) of Udder Half Defect from Day 7 to 42 in Lactation Based on Pre-Mating Udder Half Defect

The predicted probability (Pp) of udder half defect in the first six weeks of lactation (Day 7 to Day 42) based on pre-mating udder half defect status is summarized in Table 5. For udder halves that were normal at pre-mating, the Pp of an udder half remaining normal was high (Pp range 0.674–0.986) at all six events. For those that were categorised as normal at pre-mating, the Pp of being categorised as hard was highest on Day 7, before it dropped close to zero for the next four weeks and then rose again on Day 42. Whereas the Pp of an udder half being categorised as a lump was low on Day 7, peaked on Day 14, and then declined.

Only three udder halves were categorised as hard at pre-mating but the Pp of them being normal on Day 7 was very high, then declined while the Pp of being categorised as hard declined over lactation. For those that were categorised as hard at pre-mating, the Pp of being categorised as a lump was very low on Day 7 and was then higher in the subsequent weeks.

For udder halves categorised as lumps at pre-mating, the Pp of being normal increased with days in lactation, until Day 28, before declining in the following two weeks. For those udder halves that were categorised as lumps at pre-mating, the Pp of being categorised as hard was very high on Day 7 and then decreased from Day 14 while the Pp of being categorised as a lump was variable over time but was highest on Day 42.

All three udder halves categorised as hard at pre-mating expressed no milk during the first 42 days in lactation, but due to low numbers, this was not significant (*p* < 0.05). Udder halves categorised as lumps at pre-mating were four times more likely (*p* < 0.05) not to express milk, compared to udder halves categorised as normal at pre-mating.

## 4. Discussion

The objectives of the study were to assess the changes in udder half defects over time and determine the risk of future occurrence of udder half defects in non-dairy ewes. For this purpose, across two studies, a standardized udder assessment method was implemented at pre-mating, pre-lambing, docking, and weaning for two years or weekly in the first six weeks of lactation. The assessment was undertaken at the udder half level as each mammary gland is anatomically and/or physiologically independent. Lasagna plots were used to present the data as it enabled easy visualisation of changes in the udder half category over time.

In Study A, which evaluated udder halves at eight time points over two years, most udder halves were categorised as normal, while only a low percentage of udder halves were categorised as hard or lump, matching previous studies [4,18,21] when time-specific evaluations have been undertaken. However, the relatively large number of ewes included in the study still meant that meaningful analysis could be undertaken. Most udder halves categorised as having defects (either hard or lump) changed in category over time, while a small percentage of new defective udder halves (i.e., from udder halves previously categorised as normal) were observed at each time point. This approach demonstrated the dynamic nature of udder half defects and helps explain why repeated examinations of the same flock have previously resulted in different percentages of udder defects [4,12].

The proportion of udder halves categorised as hard was higher at docking (Study A) or the first couple of weeks in lactation (Study B). Similarly, a higher occurrence of hard udder or other udder defects shortly after lambing has been reported by others [20,29,30,31] and could be due to the peri-parturient relaxation of immunity which makes the udder susceptible to infection [32,33,34]. The occurrence of udder halves categorised as hard was also higher at pre-mating. The post-lactation involution period has been associated with mammary gland susceptibility, increased risk of infection, or advancement of subclinical infection to clinical because of compromised mammary defences in this period [35,36]. It can also be related to the occurrence of post-weaning chronic mastitis [7].

Udder halves categorised as lumps were more commonly identified during lactation (i.e., identified at docking and weaning) compared to during the non-lactating period (i.e., identified at pre-mating and pre-lambing). This agrees with Grant, et al. [12] who reported higher palpable udder defect occurrence in lactation compared to pregnancy. Lumps or intramammary abscesses usually occur following an intramammary infection (IMI) [11], which, in the case of this study, is likely to be first observed after early post-lambing infection or complication. Higher occurrence of lumps at weaning might be associated with physiological changes in early cessation of lactation and initiation of involution that compromise mammary gland immunity, particularly in those that did not express milk [35,37].

Utilizing an objective udder assessment method and making rational culling decisions is important to improve the health and welfare of pre-weaned lambs [3,15]. This can optimally be achieved by predicting the occurrence of udder defects in the next breeding season based on udder examination prior to mating at the time of ewes’ selection (for retention or culling). Pre-mating udder examination has been suggested to be an appropriate time to identify ewes that are likely to be unsuitable for retaining in the breeding flock [4] as ewes with udder halves categorised as hard or lump at pre-mating were associated with lower lamb survival and body weight gain in pre-weaned lambs [3,4,38]. In this study, an udder half categorised as hard or lump at pre-mating was more likely to remain as hard or change to lump compared with the normal udder halves in at least one of the key management times in the same year (pre-lambing, docking, and weaning). The findings of this study support the practice of farmers culling ewes identified with defects at weaning or premating [12,17].

A comparison of the same examination periods across years (Study A) showed that the percentage of udder halves that were defective at pre-mating and docking was higher in 2018 than in 2017. Similarly, palpable udder defects during a previous lactation were associated with an increased risk of udder defects in the current lactation in ewes in the United Kingdom [12]. This is likely a function of time; in this study, ewes with udder defects were not culled, therefore, as more ewes developed udder defects over time the percentage of identified defects would have increased. Further, given the high reappearance rates in Study B and the impact on lamb performance, the results of these studies suggest that ewes with hardness or lump/s within one or both udder halves should be culled regardless of the apparent severity of the defect at the time of examination.

In ewes with a previous history of udder half defects (Study B), the presence of udder half defects during the first six weeks of lactation was high and highly variable with numerous defect-type changes. The high occurrence in early lactation could be due to high production stress stimulating compromises in mammary defence systems following lambing [32,33,34,39]. During lactation, the occurrence of udder half defect was higher and more consistent over time in those udder halves that did not express milk in comparison with udder halves from which milk was expressed. Smith, et al. [11] reported that palpable udder defects are associated with abnormal secretions or abscesses in the mammary gland while abscesses take a couple of weeks to mature before spontaneously resolving or bursting [40,41]. This could explain the high occurrence and persistency of defects over time in those udder halves that did not express milk. Others have reported that udder defects can also induce early involution which may further compromise mammary immunity [35,42]. On the other hand, mammary emptying in those udder halves that do express milk might be helpful to prevent further health complications [14].

The overall frequency of defective udder halves decreased as the days in lactation increased. Udder halves categorised as hard declined substantially, as an udder half categorised as hard was more likely (80%) to change to normal in the first five weeks of lactation. This result agrees with previous reports of a higher occurrence of “hard udder” shortly after lambing but within two to three weeks the udder appeared normal [20,31]. Although the number of udder halves categorised as hard decreased significantly over time, if an udder half was still hard on Day 35, the probability of remaining hard was very high (>90%). This could be due to considerably decreased milk production or the initiation of early involution which compromises the mammary immunity as in the case of normal involution during drying off [35]. An udder half categorised as a lump was more likely to change to normal in the first five weeks of lactation, but the probability declined as the days in lactation increased. The probability of an udder half categorised as a lump to remain lump increased for the first four weeks in lactation and started to decrease on Day 35. This might be associated with the development and maturation of an abscess that bursts a couple of weeks later [40,41]. In the first six weeks of lactation, the probability of new defect development (normal to hard or lump) was very low, and it decreased as the days in lactation increased.

Udder palpation scoring is an easy and practical method of udder assessment in ewes. Nevertheless, the technique could be relatively subjective depending on the physiological state of the udder (i.e., lactating or involuted) and the operators’ skill. To minimize potential variation or misclassifications, in Study A, the same four trained operators undertook the udder palpations while in Study B the same person undertook all udder palpations. However, the outcomes may have been missed, misclassified, or over-interpreted, and therefore, results should be interpreted with this in mind.

In conclusion, udder half defect status changed over key management times such as pre-mating, pre-lambing, docking, and weaning in a year, and also weekly in early lactation. The highest occurrence of udder halves categorised as hard was observed during the non-lactating period and early lactation (pre-mating or docking) whereas in udder halves categorised as lump, the highest occurrence was during lactation (docking and weaning). Udder half defects categorised as hard or lump at pre-mating were more likely to be defective (hard or lump) at future examinations at key management times in the year or pre-mating of the following year. Udder halves that did not express milk in early lactation had a higher occurrence and persistency of udder half defects compared with those that did express milk. Therefore, it is recommended that farmers identify and cull ewes with udder halves categorised as hard and lump. However, further consideration should be given to the dynamic pattern of udder half defect changes over time and their persistency, the status of the contralateral udder half, and the ultimate effect on whole udder milk production.

## Figures and Tables

**Table 1:** Relative risk ratio (RRR ± standard error) of udder half defect category (normal, hard, or lump) at pre-lambing, docking, and weaning based on pre-mating udder half defect category from 1962 udder halves (from 981 non-dairy Romney ewes) in 2017 (Study A).

Predictor Variables	Category	Udder Half Defect (2017)	
		Hard	Lump
			pre-lambing
Pre-mating udder half defect	Normal (Reference)	-	-
	Hard	52.9 (0.9)	13.8 (0.4)
	Lump	26.5 (1.2)	6.9 (0.6)
Constant		0.002 (0.6)	0.002 (0.1)
			docking
Pre-mating udder half defect	Normal (Reference)	-	-
	Hard	14.1 (0.6)	1.01 ^NS^ (1.0)
	Lump	42.0 (0.55)	5.5 (0.65)
Rearing rank	Single (Reference)	-	-
	No lambs	1.8 ^NS^ (0.6)	3.6 (0.5)
	Twins	0.2 (0.65)	1.7 ^NS^ (0.3)
	Triplets	3.0 ^NS^ (0.8)	1.9 ^NS^ (0.8)
Constant		0.01 (0.3)	0.02 (0.3)
			weaning
Pre-mating udder half defect	Normal (Reference)	-	-
	Hard	43.3 (0.6)	3.3 ^NS^ (0.6)
	Lump	26.0 (0.7)	6.7 (0.5)
Constant		0.01 (0.3)	0.04 (0.1)

Note: All relative risk ratios (RRR) were significant (*p* < 0.05), except those designated as ^NS^ (not significant, *p* > 0.05).

**Table 2:** Relative risk ratio (RRR ± standard error) of udder half defect category (normal, hard, or lump) at pre-lambing, docking, and weaning based on pre-mating udder half defect category from 1538 udder halves (from 769 non-dairy Romney ewes) in 2018 (Study A).

Predictor Variables	Category	Udder Half Defect (2018)	
		Hard	Lump
			pre-lambing
Pre-mating udder half defect	Normal (Reference)	-	-
	Hard	1465 (1.1)	1462 (1.0)
	Lump	66.6 (1.4)	333 (1.1)
Constant		0.001 (1.00)	0.001 (0.9)
			docking
Pre-mating udder half defect	Normal (Reference)	-	-
	Hard	292.5 (0.7)	169.3 (0.6)
	Lump	6.2 (0.8)	16.7 (0.5)
Rearing rank	Single (Reference)		
	No lambs	8.3 (0.8)	7.4 (0.5)
	Twins	0.2 (0.84)	0.7 ^NS^ (0.5)
	Triplets	0.9 ^NS^ (1.2)	1.3 ^NS^ (0.9)
Constant		0.009	0.01 (0.4)
			weaning
Pre-mating udder half defect	Normal (Reference)	-	-
	Hard	84.7 (0.7)	76.0 (0.5)
	Lump	9.4 (1.1)	20.8 (0.5)
Constant		0.006 (0.3)	0.02 (0.19)

Note: All relative risk ratios (RRR) were significant (*p* < 0.05), except those designated as ^NS^ (not significant, *p* > 0.05).

**Table 3:** Predicted probability (Pp) of udder half defect occurrence in non-dairy Romney ewes at pre-lambing, docking, and weaning based on pre-mating udder half defect category from 1962 udder halves (981 ewes) in 2017 and 1538 udder halves (769 ewes) in 2018 (Study A).

Year	Pre-Mating Udder Half Defect Category	Change of Udder Half Defect Category	Pre-Lambing	Docking	Weaning
2017	Normal	Normal to Normal	0.987	0.968	0.959
		Normal to Hard	0.002	0.012	0.005
		Normal to Lump	0.024	0.020	0.036
	Hard	Hard to Normal	0.821	0.834	0.727
		Hard to Hard	0.036	0.149	0.182
		Hard to Lump	0.143	0.017	0.091
	Lump	Lump to Normal	0.797	0.608	0.714
		Lump to Hard	0.060	0.323	0.107
		Lump to Lump	0.143	0.069	0.179
2018	Normal	Normal to Normal	0.999	0.969	0.973
		Normal to Hard	0.001 *	0.010	0.001 *
		Normal to Lump	0.001 *	0.020	0.021
	Hard	Hard to Normal	0.333	0.160	0.320
		Hard to Hard	0.333	0.440	0.160
		Hard to Lump	0.333	0.400	0.520
	Lump	Lump to Normal	0.786	0.630	0.703
		Lump to Hard	0.036	0.074	0.001 *
		Lump to Lump	0.178	0.296	0.296

Note: * indicates probability of 0.001 or less. A Predicted probability (Pp) of 0 indicates the impossibility of the defect category occurring, and 1 indicates certainty.

**Table 4:** Predicted relative risk ratio (RRR ± standard error) of 2018 pre-mating udder half defect occurrence based on 2017 pre-mating and weaning udder half defect status from 1408 udder halves (from 704 non-dairy Romney ewes) (Study A).

Predictor Variables	Categories	Pre-Mating Udder Half Defect (2018)	
		Hard	Lump
2017 Pre-mating udder half defect	Normal (Reference)		
	Hard	15.4 (0.6)	7.6 (0.8)
	Lump	6.6 (0.8)	13.4 (0.6)
Pre-mating BCS		0.3 (0.4)	0.4 (0.4)
Ewe age (Year born)	2013 (Reference)		
	2014	1.3 ^NS^ (0.4)	4.7 (0.6)
Constant		0.4 ^NS^ (1.3)	0.1 ^NS^ (1.4)
2017 Weaning udder half defect	Normal (Reference)		
	Hard	141.2 (0.62)	53 (0.8)
	Lump	5.9 (0.5)	15.7 (0.5)
Constant		0.011 (0.2)	0.011 (0.2)

Note: All Relative Risk Ratios were significant (*p* < 0.05), except those designated as ^NS^ (not significant, *p* > 0.05).

**Table 5:** Predicted probability (Pp) of udder half defects change over time from Day 7 to 42 of lactation based on pre-mating udder half defect category in 92 udder halves (from 46 non-dairy Romney ewes; Study B).

Udder Half Defect Category		Day 7	Day 14	Day 21	Day 28	Day 35	Day 42
Pre-Mating	Lactation						
Normal	Normal	0.864	0.674	0.904	0.986	0.961	0.883
	Hard	0.113	0. 107	0.037	0.001 *	0.001 *	0.067
	Lump	0.023	0.220	0.060	0.014	0.039	0.050
Hard ^ǂ^	Normal	0.907	0.001 *	0.674	0.297	0.001 *	0.001 *
	Hard	0.093	0.001 *	0.001 *	0.001 *	0.001 *	0.001 *
	Lump	0.001 *	0.999	0.326	0.999	0.999	0.999
Lump	Normal	0.574	0.708	0.804	0.923	0.616	0.500
	Hard	0.396	0.090	0.021	0.001 *	0.001 *	0.056
	Lump	0.030	0.201	0.175	0.077	0.384	0.444

Note: The Predicted probability of each udder half defect category in the six examinations during lactation (Day 7 to Day 42) was undertaken based on the pre-mating udder half defect status of the ewes. ^ǂ^ Only three udder halves were categorised as hard at pre-mating. * indicates probability of 0.001 or less. A Predicted probability (Pp) of 0 indicates the impossibility of the defect category occurring and 1 indicates certainty.

## Data Availability

The data utilised by this study are available on request from the corresponding author.
